# Dielectric Properties of Isotactic Polypropylene with Lignocellulose-Based Biomass Filler

**DOI:** 10.3390/ma18071657

**Published:** 2025-04-04

**Authors:** Dragana D. Cerovic, Ivan M. Petronijevic, Filip S. Marinkovic, Slavica B. Maletic, Dusan M. Popovic

**Affiliations:** 1 Academy of Technical and Art Applied Studies Belgrade, Starine Novaka 24, 11000 Belgrade, Serbia; 2 University of Belgrade-Faculty of Physics, Studentski Trg 12, 11000 Belgrade, Serbia; filip@ff.bg.ac.rs (F.S.M.); sslavica@ff.bg.ac.rs (S.B.M.); drdmpopovic@gmail.com (D.M.P.)

**Keywords:** biomposites, dielectric properties, iPP, lignocellulosic biomass filler

## Abstract

The ecological aspect of substituting synthetic materials with natural materials is of great interest nowadays. This paper examines the percentage of lignocellulose-based fillers that can be added to a synthetic polymer matrix to ensure the resulting biocomposite maintains its dielectric properties. Biocomposites were made from isotactic polypropylene (iPP) and various proportions (20%, 30%, and 40%) of oats, rye, wheat, and barley bran and granules from corn cobs using a Brabender plastograph and a hydraulic hot press. From a morphological analysis, it was noted that the particles were well incorporated into the polymer matrix. The frequency-dependent behavior of the dielectric properties was analyzed across a frequency range from 30 Hz to 60 kHz at a room temperature of 23 °C and 35% relative humidity. The obtained results showed that the incorporation of biomasses into the iPP matrix increased the values of the dielectric properties across the entire measured frequency range. The samples with wheat showed the most stable values of the dielectric parameters with frequency changes, for all three concentrations. A linear regression analysis showed a very high coefficient of determination (R^2^ = 0.997) between the effective dielectric permeability and filler concentration at 30 Hz for the samples with wheat. Furthermore, the biocomposite iPP/20% wheat showed a desirable balance of dielectric properties for electronic applications. The results showed that biocomposites obtained by adding cheap lignocellulose-based biomass, such as bran or granules from corn cobs, to a synthetic polymer matrix have a great potential for use as electrically insulating materials because their dielectric parameters are comparable to those of standard insulating materials.

## 1. Introduction

The concept of sustainable development for the preservation of nature determines the need for the design of eco-friendly materials as a substitute for the synthetic polymeric materials which are increasingly being used. The increasing problem of environmental pollution sets the task of developing environmentally friendly materials from renewable resources that can be easily destroyed or recycled. Biocomposites obtained from biodegradable materials, such as lignocelluloses, and synthetic polymers are very interesting for further use, both from an economic and an environmental point of view [1,2,3]. Lignocellulosic biomass is non-toxic and easily available because it comes from renewable sources, is biodegradable, has a low specific gravity, neutrality to carbon dioxide emission, has no health risks, and is low cost. Lignocellulosic materials comprise forestry, agricultural, and agro-industrial wastes, which include various materials such as sawdust, waste paper, grass, straw, stems, stalks, leaves, husks, and husks from cereals such as rice, wheat, corn, and barley [1]. The combination of lignocelluloses and synthetic polymers creates the ability to develop diverse products for different purposes. Biocomposite materials have a wide range of uses for consumer goods, as well as for automotive, transportation, construction, and packaging applications [4,5]. Inexpensive agricultural biomasses, like the bran obtained from the grain of oats, wheat, rye, and barley, have a great potential for use in sustainable bio-based composite materials. Bran (or cereal husks) is generated as a bio product during the milling of grain. The accumulation in nature of this abundant, renewable, and inexpensive energy source constitutes the loss of a potentially valuable energy source and environmental problems caused by their accumulation in nature. As a result, interest for agricultural waste as fillers in polymers has been growing.

Broadband dielectric spectroscopy (BDS) is a rapid and non-destructive measuring technique that provides information about the dielectric response of materials to electric fields of different frequencies. Knowledge of the relationship between frequencies and dielectric properties is helpful in determining the optimum frequency range in which the material has the desired dielectric characteristics for the intended applications [6]. The dielectric properties of the material provide valuable information about the storage and dissipation of electric fields in materials. Therefore, they provide insights into the feasibility of using the material for electrical insulation applications. The response of a dielectric material to an applied electric field usually is described by the relative dielectric permeability (*ε*′), dielectric loss tangent (*tanδ*), and dielectric loss factor (*ε*″). Relative dielectric permeability is a measure of the amount of energy from an external electrical field stored in the material, while the loss factor is a measure of the amount of energy loss from the material due to an external electric field. The dielectric loss tangent is the ratio of the lost energy to the stored energy per cycle of the applied external field.

Testing the dielectric properties of agricultural materials is important for two types of applications: the dielectric heating of materials and quality sensing [7]. In the agricultural industry worldwide, the dielectric properties of grains and seeds are used for the rapid sensing of moisture content [8,9] or the indication of some other quality attributes [7]. Numerous studies [9,10,11] on different kinds of grains and seeds showed that the dielectric properties of these materials vary with the frequency of applied electric field, the temperature, the moisture content of these materials, their structure and constituents, and their bulk density. Numerous studies conducted by Nelson [12,13,14] and his co-authors have shown a decrease in the dielectric permeability values of grain and seed samples with increasing frequency, and an increase with increasing humidity. The results obtained show a similar frequency-dependence of dielectric properties for wheat and oats samples [12], as well as for corn, wheat, barley, and oats [13] in a wide frequency range. Nelson and Trabels [15] developed models to predict the dielectric properties of several important grains and oilseeds at 23 °C. They analyzed the microwave dielectric properties of wheat, corn, barley, oats, sorghum, soybeans, canola, shelled peanuts, and peanut pods, and based on the nearly linear relationship between the dielectric properties and the log of frequency or frequency as well as moisture content, they developed multiple linear regression models to predict dielectric properties.

In recent years, there has been an increased interest in developing dielectric materials from agricultural-waste-reinforced polymeric composites for use in electrical and electronic power systems [16,17,18,19]. Lignocellulose-based raw materials are used in many electrical insulation applications due to their low cost, low weight, availability, and biodegradability, as well as good electrical and mechanical properties. However, since these materials are extremely hygroscopic, they would act effectively as electrical insulators in the lower humidity operating environment. Analyzing the electrical insulation properties of seven samples: shells of coconut, mango endocarp, palm kernel, groundnut, and bean, as well as corncob and rice husk bound with gum arabic, Inegbenebor and Adeniji [16] showed that these materials had electrical insulative properties comparable with the known standard values. Rajamanikandan et al. [17] investigated the chemically treated banana leaf fiber-reinforced epoxy composites for high-voltage electrical insulation applications at different temperatures and frequencies. The dielectric properties of banana leaf fiber epoxy composites increase with the weight percentages of banana fiber loading while decreasing with increased frequency from 1 kHz to10 KHz. Changes in the values of dielectric properties with increasing temperature and frequency are related to orientation polarization and interfacial polarization.

This work aimed to obtain materials suitable for electrical insulation by adding different concentrations of lignocellulose-based biomass fillers to a non-degradable polymer matrix. Biocomposites were designed by adding different percentages of bran and granules from corn cobs (Cellgran) to the polymer matrix of isotactic polypropylene, and their dielectric properties were investigated as a function of the frequency from 30 Hz to 60 kHz at room temperature. Isotactic polypropylene was blended with various proportions of oats, rye, wheat, barley, and Cellgran (20%, 30%, and 40%). An analysis of the relative change in the value of the dielectric parameters concerning the value of the polymer matrix due to the addition of filler was performed. It was also considered which sample showed the desired balance of dielectric properties for electronic applications.

## 2. Materials and Methods

### 2.1. Materials

Different biocomposites (15 samples) were produced by the introduction of lignocellulose-based fillers into a polymer matrix in different percents. Isotactic polypropylene (iPP) in a granule form for the matrix was obtained from Hipol, Odzaci, Serbia. The data of this commercial polymer are HIPOLEN P EH71, *M_w_* = 110,000 g/mol, *ρ* = 0.9 g/cm^3^, crystalinity 35%, and *T*_g_ ≈ 0 °C. Polymer iPP in the form of granules is suitable for compression molding and twin-screw-blade-heating Measuring Mixers. This makes it appropriate for matrices for the preparation of composites.

Regarding the lignocellulose-based fillers, the bran was produced in Serbia from wheat by Unimiler, Belo Polje; oats by Italico, Becej; and rye and barley by Shaula, Stari Banovci. The Cellgran^®^ C was produced by the Maize Research Institute “Zemun Polje” (Belgrade, Serbia). Celgran products are lignocellulose granules obtained by the processing of maize cobs according to the original technology by the Maize Research Institute “Zemun Polje”, Belgrade, Serbia [20,21].

### 2.2. Sample Preparation

The sample preparation was started by grinding bran and Cellgran granules for 5 min in a grinder, and the obtained particles were of different sizes. After that, the ground bran and Cellgran were dried in an oven at 80 °C for 24 h prior to processing to remove moisture. The iPP was blended with various proportions of ground bran and Cellgran (20%, 30% and 40%) using a Brabender plastograph (Brabender EC plus Measuring Mixer, Duisburg, Germany) operating at 170 °C for 10 min at 70 rpm. First, only the iPP was put in the plastograph, and after 3 min, lignocellulosic fillers were added. The pure iPP and iPP/bran composite samples were then removed from the plastograph as small clumps and molded at 175 °C for 3 min in a hydraulic hot press (Polystat Press 200T, Servitec, Wustermark, Germany). After cooling in the air, the samples were cut in the shape of discs that were 2 mm in thickness and 27 mm in diameter.

### 2.3. Methods

The morphological characterization of the cross-section of the biocomposite samples was conducted on a scanning electron microscope (SEM), JEOL, JSM-840A Scanning Microscope, Tokyo, Japan, at an acceleration voltage of 20 kV. Micrographs with a magnification of 400× were obtained. A Nano Measurer 1.25, Jie Xu, the Department of Chemistry, Fudan University, was used for the particle distribution determination.

Dielectric spectroscopy measurements were performed on a Hameg 8118 (Programmable LCR bridge Hameg 8118, Rohde and Schwarz, Munich, Germany) instrument at 14 frequencies in the range from 30 Hz to 60 kHz at a room temperature of 23 °C and a relative humidity of 35%. The spacing of the electrodes (*d*) of the capacitance cell was controlled by a micrometer. The values of the capacitance of the capacitor with the dielectric samples (*C*), and the capacitance of the capacitor without the dielectric samples (*C*_0_) but with space between electrodes equal to the thickness of the sample, were registered in the parallel capacitance mode of the instrument. The signal voltage required to generate the electric field was set to 1.5 V.

Based on recorded values, effective dielectric permeability was calculated according to the following equations:

(1) $$\varepsilon_{m}^{\prime }=\frac{\left(C-C_{0}\right)}{\varepsilon_{0}\;\cdot \;S}\cdot d+1$$

where *ε*_0_ is the dielectric permeability of the free space *ε*_0_ = 8.85·10^−12^ F·m^−1^, and *S* is the area of the sample. Three measurements were performed for each sample type.

## 3. Results

### 3.1. Morphological Characterization

**Figure 1:**
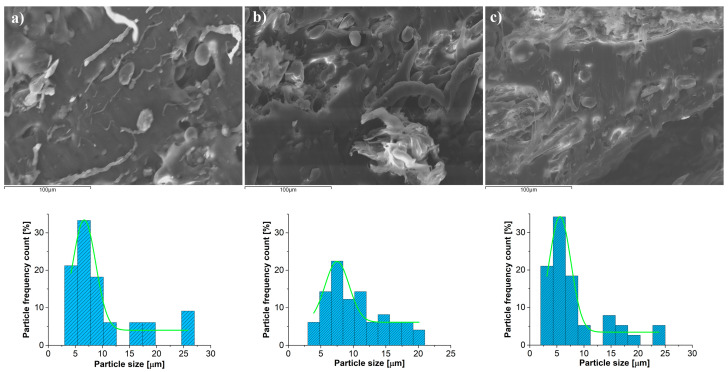
SEM micrographs of biocomposite cross-sections with particle size distribution histogram: (**a**) 20% wheat + 80% iPP; (**b**) 30% wheat + 70% iPP; and (**c**) 40% wheat + 60% iPP.

In Figure 1, a micrograph cross-section of the biocomposite with wheat (20% wheat + 80% iPP; 30% wheat + 70% iPP; and 40% wheat + 60% iPP) recorded by SEM is shown.

The morphological properties of prepared biocomposites (size, shape, and particle size distribution) are considered. A cross-sectional SEM image showed rough, irregularly shaped structures and voids (darker areas). Two different kinds of particles are observable. The ellipsoidal or egg-shaped particles, with a smooth and clean surface, are the first kind. These particles are starch granules. The second type of particles are irregularly shaped particles. These particles are starch granules with remnants of the protein matrix. Histograms with Gaussian fit for the corresponding cross-sections are also presented in Figure 1. An analysis of the particle sizes with the Nano Measurer showed the narrow distribution of particles up to 21.6 µm wide for the sample 20% wheat + 80% iPP, 16.2 µm for the sample 30% wheat + 70% iPP, and 20 µm for the sample 40% wheat + 60% iPP. All the samples show a narrow Gaussian peak, and it is shifted toward smaller diameter particle sizes. Most of the particles are between 4 µm and 8 µm in diameter. The agglomeration of particles is not observed. It can be noted that the particles are well incorporated into the polymer matrix. Also, the distribution and dispersion of the particles in the biocomposites are satisfactory.

### 3.2. Change in Effective Dielectric Permeability

**Figure 2:**
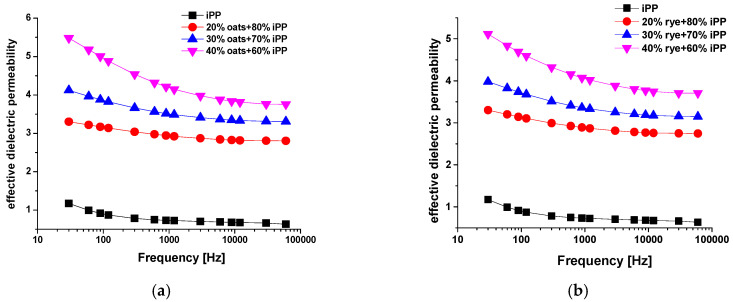

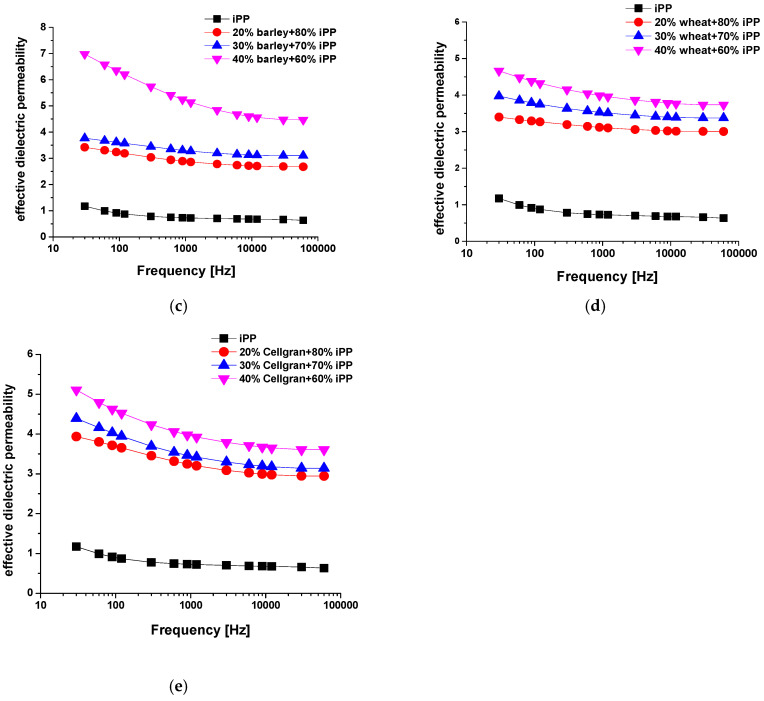
Frequency dependence of effective dielectric permeability of pure iPP and biocomposites of iPP with 20%, 30%, and 40% (**a**) oats, (**b**) rye, (**c**) barley, (**d**) wheat, and (**e**) Cellgran.

Experimental effective dielectric permeability values ($\varepsilon_{m}^{\prime }$) as a function of the frequency are presented in Figure 2, for the biocomposites of isotactic polypropylene (iPP) with oats, rye, wheat, barley, and Cellgran. In order to compare, the values and the frequency dependence of the dielectric permeability for the pure iPP are also shown on each figure. The obtained results showed that the incorporation of biomass fillers into the polymer matrix increased the values of the dielectric permeability in the entire frequency range, which is in agreement with the literature [22,23]. The increase occurs due to the presence of polar groups in the lignocellulose-based fillers. The insertion of polar groups into a non-polar iPP, which shows only current ionic and electronic polarization, leads to the appearance of polarization related to dipole reorientation in an applied field [24]. Moreover, due to the differences in the conductivity of the polymer matrix and the filler in the composite materials, interfacial polarization, also known as Maxwell–Wagner–Sillars polarization (MWS), also occurs [25].

A non-polar polymer, such as iPP, has values of dielectric permeability that remain nearly constant over the whole measuring frequency range [26,27]. On the other side, with the increase of frequency, effective dielectric permeability values of the samples with 20%, 30%, and 40% lignocellulose-based fillers have a monotone decreasing trend. This trend of decreasing effective dielectric permeability values with the increasing frequency of an applied field occur due to the impossibilities of the dipoles to self-rotate with the direction of the applied field at higher frequencies. At low applied frequencies, the effective dielectric permeability values were shown to decrease slightly with increasing frequency until it reached 6 kHz and remained relatively stable between 6 kHz and 60 kHz for the samples with 20% and 30% biomass fillers. This is evident from Figure 2.

The more prominent decrease values of effective dielectric permeability with the increasing frequency were recorded for samples with 40% fillers. The change of the value of effective dielectric permeability, ${\Delta \varepsilon }_{m}^{\prime }=\varepsilon_{30\;Hz\;}-\;\varepsilon_{60\;kHz}$, occurred in the samples with barley 2.51, oats 1.72, Cellgran 1.5, rye 1.40, and wheat 0.93. The smallest change in the effective dielectric permeability value with the increasing frequency for the samples with wheat was also noted in work [13].

It is evident from the results that the bio-based samples show similar dielectric behavior with a change in frequency at room temperature, which indicates their identical molecular setup. The frequency dependence of the dielectric permeability trend agrees with the trend obtained for different varieties of wheat and oats at low moisture contents [12,28], as well as for corn, wheat, barley, and oats [13].

**Figure 3:**
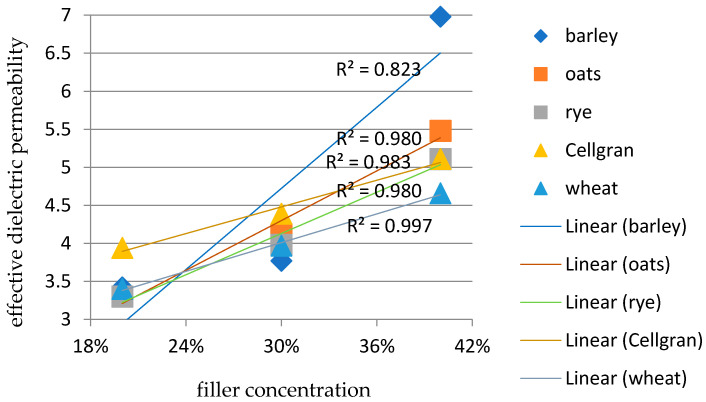
Correlation between the filler concentration and values of effective dielectric permeability at 30 Hz for biocomposites of iPP matrix with barley, oats, rye, Cellgran, and wheat.

With an increase in the weight percentage of fillers in the iPP matrix, the values of the effective dielectric permeability also increases for all the varieties of fillers used due to the increase in the number of polar groups, as well as by increasing the interphase surface between the matrix and the filler. The dielectric permeability is higher due to polarization exerted by the incorporation of lignocellulosic fibers [24,29]. According to the linear regression analysis (Figure 3) between the effective dielectric permeability and filler concentration at 30 Hz, the greatest change in the effective dielectric permeability values was recorded for the sample with barley (the coefficient of determination R^2^ was 0.823), while the most stable value obtained was for the sample with wheat: the R^2^ was 0.997. For the other samples, the coefficient of determination was approximately 0.983 for the sample with Cellgran, and it was 0.980 for the samples with rye and oats.

**Figure 4:**
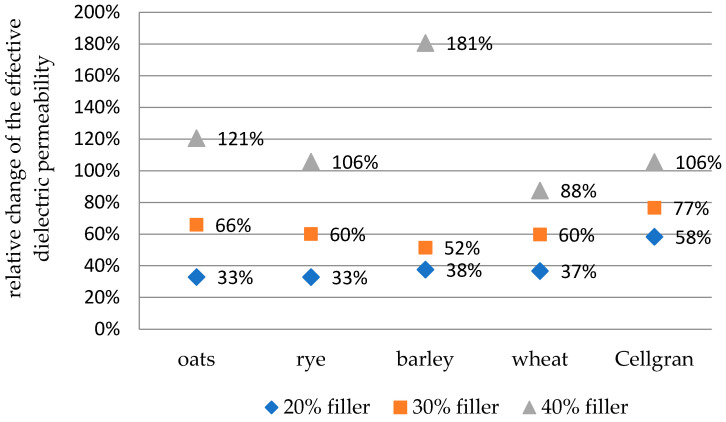
The values of the relative change in the effective dielectric permeability with the addition of 20%, 30%, and 40% biomass fillers compared to pure iPP at 30 Hz.

**Figure 5:**
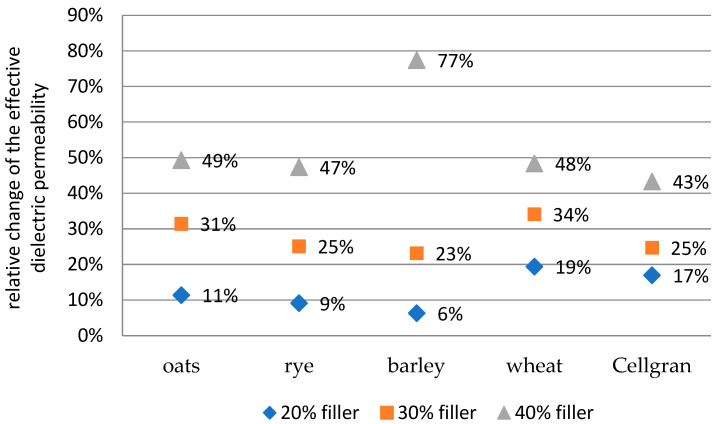
The values of the relative change in the effective dielectric permeability with the addition of 20%, 30%, and 40% biomass fillers compared to pure iPP at 60 kHz.

The values of the relative change in the effective dielectric permeability with the addition of 20%, 30%, and 40% biomass fillers compared to the pure iPP are shown for the results at 30 Hz in Figure 4 and at 60 k Hz in Figure 5. The relative change was calculated according to the formula

(2) $$\delta_{1}=\frac{\varepsilon_{biocomposit-\;}\varepsilon_{ipp}}{\varepsilon_{ipp}}\;$$

At a frequency of 30 Hz, the largest increase in the effective dielectric permeability with an addition of 20% and 30% fillers was shown by the samples with Cellgran: 58% and 77%, respectively. Samples with 20% added bran showed almost the same relative increase in the effective dielectric permeability compared to the iPP polymer matrix: 33% for oats and rye, 37% for wheat, and 38% for the samples with barley. A slightly larger change was obtained for the samples with 30% added bran: 52% for barley, 60% for rye and wheat, and 66% for the samples with oats. The largest change compared to the pure iPP, of 181%, was recorded for the sample with 40% barley bran. The samples with 40% added oats showed a 121% increase, those with rye and Cellgran showed a 106% increase, and the smallest change was for the samples with wheat: 88%.

For samples with 20% and 30% wheat, slight increases of 19% and 34%, respectively, were observed at a frequency of 60 kHz (Figure 5). Further, the change was slightly smaller (6%, 9%, 11%, and 17%) for the other samples with 20% barley, rye, oats, and Cellgran, respectively. For the samples with 30% barley and oats, the increases were 23% and 31%, respectively, and it was 25% for the samples with Cellgran and rye.

A more significant increase of 77% of the effective dielectric permeability value was shown by the sample with the 40% addition of barley in the iPP matrix. Furthermore, the other samples showed smaller values of the relative increase in the effective dielectric permeability value compared to the value of the iPP matrix: 49%, 47%, 48%, and 43% for oats, rye, wheat, and Cellgran, respectively.

In order for the material to be suitable for insulation, it is necessary to have a low dielectric permeability. The highest dielectric permeability value obtained was 6.98 for the samples with barley, which was about 2.7 times higher than the iPP matrix. It can be stated that even with an increased percentage of filler, there was no significant increase in the results and that the material still remains able to be used as an insulator.

### 3.3. Change in Dielectric Loss Tangent

**Figure 6:**
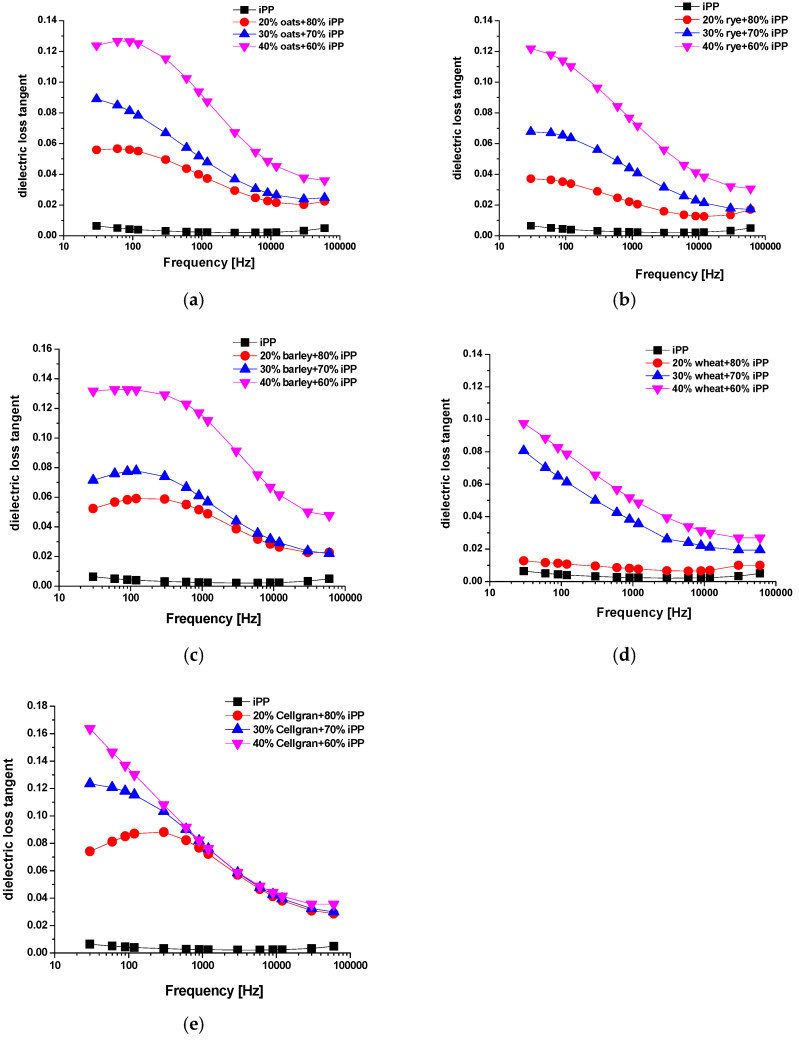
Frequency dependence of dielectric loss tangent of pure iPP and biocomposites iPP with 20%, 30%, and 40% (**a**) oats, (**b**) rye, (**c**) barley, (**d**) wheat, and (**e**) Cellgran.

The dielectric tangent of losses correspond with energy losses, which happen due to the motion or rotation of atoms or molecules within the dielectric sample positioned in a periodic electric field. Figure 6 shows the dielectric spectra of the dielectric loss tangent (*tanδ*) of the iPP sample and the samples with biomass fillers from 30 Hz to 60 kHz. The values of the dielectric loss tangent remain nearly constant over the whole frequency range for the sample of pure iPP, which is in good agreement with the results obtained in [26,27]. The low polarity of iPP, low hygroscopicity, and low content of contaminants introduced during the synthesis causes low values of dielectric loss tangent and dielectric permeability in a wide range of frequencies.

For the samples with biomass fillers, the frequency dependence of the dielectric loss tangent was found to be less regular than the effective dielectric permeability over the measured frequency range. Similar trends are quoted for hard red winter wheat by Nelson [28] and for flaxseed by Sacilik et al. [11]. In addition, the results showed that the values of the dielectric tangent loss also increased with the increasing weight percentage of the biomass filler loadings, and that the effect was predominant at lower frequencies [25]. The changes in the values of the dielectric loss tangent with the increasing frequency were more intense in the samples with higher filler contents. The same trend was observed for effective dielectric permeability. The wide and weak peak was recorded in the measuring frequency range for the samples with fillers of oats, rye, barley, and Cellgran, respectively. The wide transition recorded in the measuring frequency range can be related to the dielectric relaxation and dispersion phenomena. In the samples with Cellgran, relaxation was recorded only for the samples with 20% filler, while relaxation was completely absent in all the samples with wheat.

For the samples with 20% and 30% oats, rye, and barley, the change values of the dielectric loss tangent were negligible above the frequencies 6 kHz, 30 kHz, and 3 kHz, respectively. The samples of wheat and Cellgran with all of the three weight percentages had a negligible change above the frequencies of 6 kHz and 0.6 kHz, respectively. At lower frequencies, the dielectric loss values could be related to space charge polarization (MWS), i.e., by the accumulation of charges at the interfacial of the filler and the polymer matrix.

**Figure 7:**
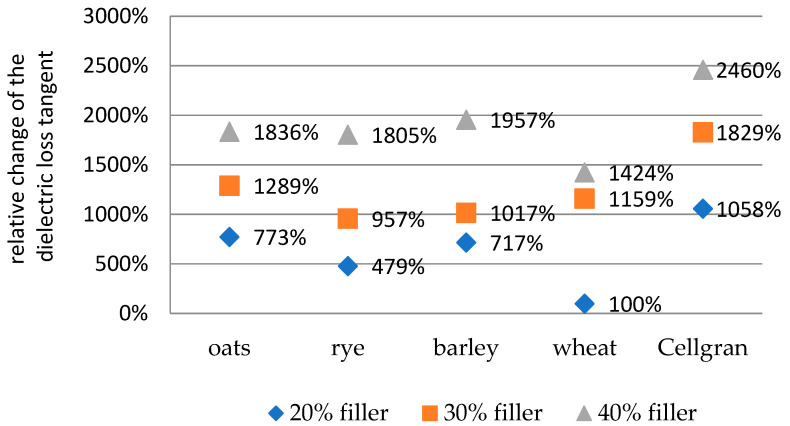
The values of the relative change in the dielectric loss tangent with the addition of 20%, 30%, and 40% biomass fillers compared to pure iPP at 30 Hz.

**Figure 8:**
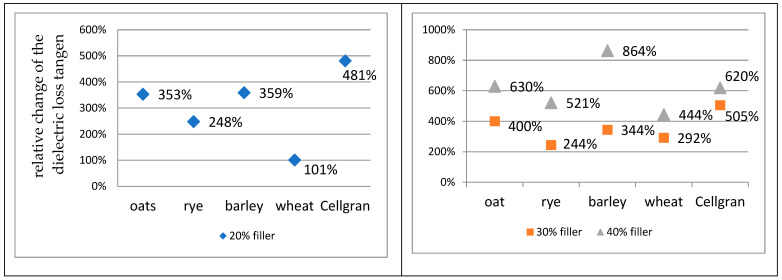
The values of the relative change in the dielectric loss tangent with the addition of 20%, 30%, and 40% biomass fillers compared to pure iPP at 60 kHz.

The values of the relative change in the dielectric loss tangent with the addition of 20%, 30%, and 40% biomass fillers compared to sample of pure iPP are shown at 30 Hz (Figure 7) and 60 kHz (Figure 8). The relative change was calculated according to the formula

(3) $${\;\delta }_{2}=\frac{{tan\delta }_{biocomposit-\;}{tan\delta }_{ipp}}{{tan\delta }_{ipp}}$$

The relative changes in the dielectric loss tangent of the prepared biocomposites, compared to the dielectric loss tangent values of iPP, were greater than the changes in the epsilon. However, although the values of the dielectric loss tangent increased, they remained significantly lower than one in the whole measured range.

The highest dielectric loss tangent value of 0.097 was obtained for the sample with 40% Cellgran.

At a frequency of 30 Hz, the largest increase in the dielectric loss tangent for additions of 20%, 30%, and 40% was shown by Cellgran: 1058%, 1829%, and 2460%, respectively. The smallest change for 100% was observed for the sample with 20% wheat, while the relative change values of the samples with 20% added bran compared to pure iPP were 479% for rye, 717% for barley, and 773% for the samples with oats. The samples with 30% filler showed approximate relative increases of the dielectric loss tangent compared to the iPP matrix of 957%, 1017%, 1159%, and 1289% for rye, barley, wheat, and oats, respectively. Relative change in the values for the samples with 40% wheat, rye, oats, and barley were 1424%, 1805%, 1836%, and 1957%, respectively. However, the recorded values for the dielectric loss tangent were significantly lower than unity, which indicates that the preparatory compounds belong to dielectrics.

At a frequency of 60 kHz, the relative increase of the dielectric loss tangent of the prepared biocomposites compared to the polymer matrix was smaller for all the samples than the changes at 30 Hz. The prepared biocomposites contain a large number of particles of different sizes, so that the polarization of the space charge is dominant at lower frequencies, while at higher frequencies, the particles were not able to follow field changes. The smallest relative increase in the dielectric loss tangent with an addition of 20% and 40% was shown by the samples with wheat: 101% and 444%, respectively. For the sample with 30% wheat, the relative increase was 292%. The samples with 20% and 30% added rye, barley, Cellgran, and oats showed almost the same relative increase in the dielectric loss tangent compared to the polymer matrix. The relative change values for the samples with 20% added rye, barley, Cellgran, and oats were 248%, 359%, 481%, and 353%, respectively, while the values with 30% filler were 244%, 344%, 505%, and 400%, respectively. By adding 40% filler, slightly higher values were obtained. For rye, 521%, for Cellgran, 620%, and for oats, the relative change was 630%. The sample with 40% barley recorded the largest change compared to the pure iPP, 864%, while the recorded value of the dielectric loss tangent was 0.048.

Based on the obtained results, it is evident that the lignocellulose-based biomass filler has a greater influence on the trend of dielectric properties changes with increasing frequency than the iPP matrix. This is consistent with the fact that the component with the higher values of dielectric properties has a predominant role in the blend [30].

### 3.4. Optimization of the Dielectric Properties

**Figure 9:**
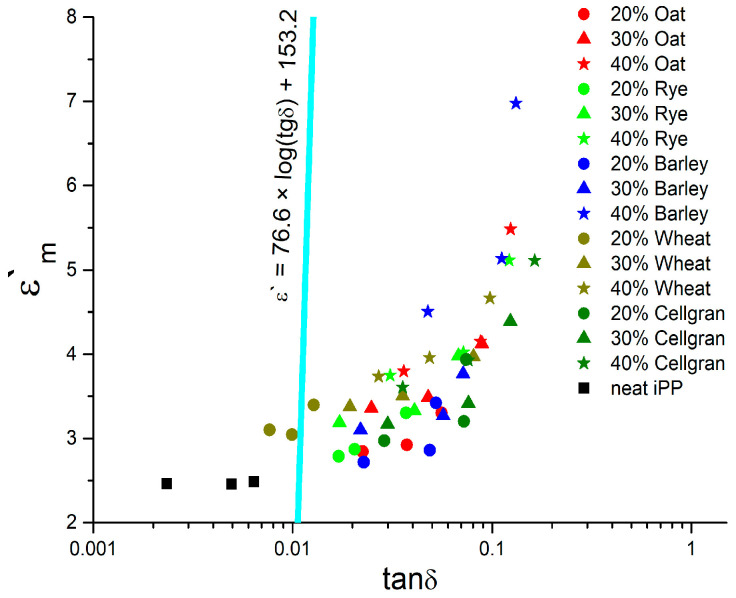
The optimization of the dielectric permeability and dielectric loss tangent at 30 Hz, 1.2 kHz, and 60 kHz for dielectric application (circle—20% biocomposite; triangle—30% biocomposite; star—40% biocomposite; black square—pure iPP; colors represent biocomposites based on oats, rye, barley, wheat, and Cellgran).

In order to obtain dielectric material with desirable dielectric properties, the values of the effective dielectric permeability (*ε_m_*′) and the values of the loss tangent (*tanδ*) should be in balance, which is important for the polymer matrix composite dielectrics. The optimal balance of these parameters for electronic applications is based on the numerous reviews and defined in previous studies by many authors [31,32,33] by the linear function *ε_m_*′ = 76.6 *log*(*tanδ*) + 153.2. The surface area above (left) this function defines the desirable balance of dielectric parameter values that justify their application for electronic devices. The diagram of dependence, *ε_m_*′ = *f*(*tanδ*), for all the samples including pure iPP (as control) at three frequencies is presented in Figure 9. The optimal types and concentrations of lignocellulose-based biomass fillers in the iPP polymer matrix for dielectric material were considered. Dielectric material for electronic application demands a low dielectric permeability and low losses. The international technology road map for semiconductors (ITRSs) indicates that future electronics will require a permeability value of 2.1 to 2.5 [34].

A well-known application of polypropylene is the polypropylene film capacitor. Values of *ε_m_* and *tanδ* for iPP justify its application according to the aforementioned function for all the measured frequencies (30 Hz, 1.2 kHz, and 60 kHz).

In Figure 9, the optimization of the dielectric permeability and the dielectric loss tangent at 30 Hz, 1.2 kHz, and 60 kHz are presented. Dot symbol shapes represent the different percentages of biomass filler, and dot symbol colors represent the type of biocomposite.

For most of the samples, the values of the loss tangent decrease with the increasing frequency, and their symbols lay on the right side of the optimal linear function *ε*′ = *f*(*tanδ*). The highest values of the loss tangent were at 30 Hz; the lower values were at 1.2 kHz; and the lowest were at 60 kHz. At the same time, the values of the dielectric permeability decrease with the increasing frequency, as shown in the previous figures of the loss tangent and permeability spectra. It can be noted that the neat iPP symbol is lying on the left side of optimal function. Also, the biocomposites based on iPP and 20% wheat filler show a desirable balance of the loss tangent and the dielectric permeability, since the two symbols are lying on the left side of the function, and one dot is very close to this line (at 30 Hz). Also, the biocomposite iPP/20% rye dots are grouped very close to the optimal function at the right side, at 60 kHz and 1.2 kHz.

## 4. Conclusions

The increasing amount of agricultural waste or by-products is a huge environmental problem that can be solved by applying them as fillers in polymers to prepare novel materials with unique properties. The motivation for this work was to examine the percentage of synthetic polymer that can be replaced by natural materials, such as fillers from lignocellulose-based biomass, in order to obtain a suitable material for electrical insulation applications. For that purpose, isotactic polypropylene was blended with various proportions (20%, 30%, and 40%) of oats, rye, wheat, and barley bran and granules from corn cobs (Cellgran). A morphological characterization showed that the particles were well incorporated into the polymer matrix. An analysis of the distribution and dispersion of the particles in the biocomposites was satisfactory.

This study’s outcome indicates that the values of dielectric properties (effective dielectric permeability and dielectric loss tangent) increased with the addition of biomass fillers, as well as with an increase in the weight percentage. This increase is more dominant at low frequencies. The increase in the values of dielectric parameters occurs due to the rise in the number of polar groups present in lignocellulose-based materials, which resulted in orientation polarization. Also, interfacial polarization also occurs due to the differences in the conductivity of the matrix and the filler in the composite materials. The dielectric properties increase with the biomass fillers’ loading at all frequencies. Furthermore, it was found that increasing the frequency provoked a decrease in the dielectric properties of the tested samples.

The analysis of the obtained results showed that with an increase in the content of the lignocellulose-based biomass in the isotactic polypropylene matrix, the effective dielectric permeability values did not exceed the values corresponding to the dielectrics. Also, the dielectric loss tangent values were significantly lower than the unity in the measured frequency range for all the prepared samples, so these materials are suitable for use as insulating materials.

Furthermore, the investigation showed that the iPP/20% wheat biocomposite exhibited a desirable balance of dielectric properties for electronic applications. Also, the iPP/20% rye biocomposite shows a potential for application with additional treatment. Further research would involve the finer tuning of the filler concentrations in order to obtain desirable properties for electronic applications.

## Data Availability

The original contributions presented in this study are included in the article. Further inquiries can be directed to the corresponding authors.
